# A Rapid Tricolour Immunochromatographic Assay for Simultaneous Detection of Tricaine and Malachite Green

**DOI:** 10.3390/bios12070456

**Published:** 2022-06-26

**Authors:** Yi-Kuan Lu, Di Xu, Wen-Yue Liu, Jing Xie, Ying Lu

**Affiliations:** 1 College of Food Science and Technology, Shanghai Ocean University, Shanghai 201306, China; m190300729@st.shou.edu.cn (Y.-K.L.); m200300932@st.shou.edu.cn (D.X.); m200300883@st.shou.edu.cn (W.-Y.L.); jxie@shou.edu.cn (J.X.); 2 Laboratory of Quality and Safety Risk Assessment for Aquatic Products on Storage and Preservation (Shanghai), Ministry of Agriculture, Shanghai 201306, China; 3 Shanghai Engineering Research Center of Aquatic-Product Processing and Preservation, Shanghai 201306, China

**Keywords:** tricolour test strip, tricaine mesylate, malachite green, simultaneous detection, Au nanoparticles

## Abstract

In this research, we designed a rapid tricolour immunochromatographic test strip with double test lines (TS-DTL) and two-colour AuNP probes, which realised the simultaneous detection of tricaine mesylate (TMS) and malachite green (MG). Through a distinct tricolour system (red T_1_ line, blue T_2_ line and purple C line), a visual identification of TMS (0.2 μg/mL) and MG (0.5 μg/mL) was quickly achieved on site, which improved the accuracy of naked eye observations. The LODs of TMS in aquaculture water, fish and shrimp were 11.0, 29.6 and 61.4 ng/mL, respectively. MG LODs were 47.0 ng/mL (aquaculture water), 82.8 ng/mL (fish) and 152.4 ng/mL (shrimp). The LOD of MG was close to the similar TS methods. However, visual detection of TMS could meet the requirements of the residue limit (1 μg/mL) of TMS in the USA, and the quantitative detection of TMS was over 16 times lower than the USA standard. The developed platform was rapid (~20 min, HPLC~3 h) and accurate, which was verified using a traditional HPLC method. The recovery rates ranged from 82.2% to 108.6% in three types of real samples, indicating a potential application in on-site fast screening or multiple detection for TMS and MG residues in aquatic products.

## 1. Introduction

With the development of high-density aquaculture transportation, an increasing number of anaesthetics are used in fisheries to keep living aquatic products in a state of low energy consumption and slowed metabolism [1]. Tricaine mesylate (TMS), a type of caine, due to its effectiveness and high metabolic rate, is widely used in living fish surgical procedures and long-distance transportation of fresh fish [2]. However, a strict limit has been set on the withdrawal time of TMS to avoid real implications for human health (USA~21 d, Canada~7 d) [3]. It has been reported that the TMS residues in fish could produce harmful effects on human health, such as endocrine disorders and altered cardiovascular function [4].

Malachite green (MG), a triphenylmethane chemical, is not only an industrial dye but also can be used in the fish farming industry. Due to its low cost and good drug effect, it has been used for years as a fungicide and parasiticide in aquaculture and fisheries [5]. However, research has shown that MG may cause teratogenic, carcinogenic and genotoxic effects in humans [6].

To reduce the risk of TMS and MG residues in the transportation and circulation of aquatic products, various analytical methods have been developed [7,8,9,10,11]. Previous research on the detection of TMS and MG has focused on instrumental analysis methods, such as high-performance liquid chromatography [7], high-performance liquid chromatography-tandem mass spectrometry [8,9], surface-enhanced Raman spectroscopy [10], and electrochemistry [11], which have the advantages of a favourable accuracy and high sensitivity. However, these methods require professional operation, expensive equipment, long experimental times and complicated sample pretreatment, which limit their application in basic laboratories or on-site detection. Hence, it is necessary to develop a sensitive, rapid and easy-to-use method for the supervision of TMS and MG.

In recent years, immunochromatographic test strip (ICTS) technology based on specific reactions between monoclonal antibodies (mAbs) and antigens has been widely applied in the fields of food safety [12] and disease diagnosis [13] due to its advantages of a simple operation, low cost and fast on-site detection. The common ICTS employs coloured or luminescent nanoparticles as detection probes, such as Au nanoparticles (AuNPs) [14], silica nanoparticles [15] and quantum dots [16]. Among them, AuNPs are the most commonly used marker for ICTS due to their good biocompatibility, visualisation and low cost. To meet the needs of simultaneous and rapid detection of multiple substances, a new ICTS technology was developed based on two or more T lines [17], an engineered strip architecture [18] or probe-coated multiple types of mAbs as detection markers [19]. In general, one T line labelling two-colour probes require a complex quantitative technique, while two or more T lines with the same nanoparticle probes may cause misreading, and the use of multiple mAb markers increases the cost of detection. Therefore, multi-T lines combined with multicolour nanoprobe markers are a research direction for the development of simultaneous, high-throughput rapid detection. Yang et al. [20] selected three-coloured latex beads (red, black and blue) as multicolour ICTS labels, realising the simultaneous measurement of three antibiotic residues in milk. Among multicolour probes, AuNPs could be ideal because of their bare-eye-detectable readout, simple synthesis, low cost and colour change based on different sizes. For example, Peng et al. [21] successfully synthesised three kinds of AuNPs with different sizes and colours (20 nm—red, 35 nm—blue, 36 nm—purple) and found that they could be applied to develop ICTS for multiple substances.

**Scheme 1:**
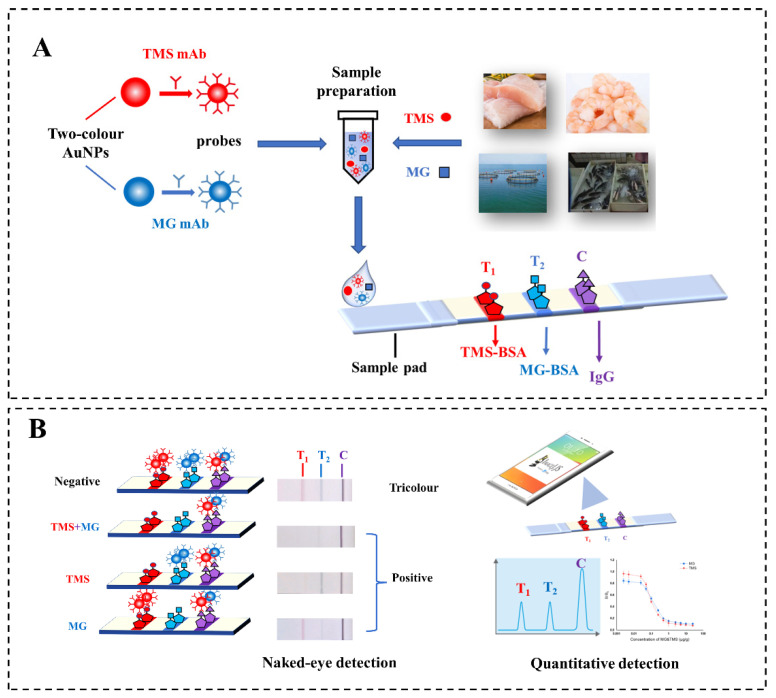
Schematic diagram of simultaneous detection for TMS and MG based on a test strip with double T lines (TS-DTL). (**A**) Schematic illustration of two-colour AuNP probes and TS-DTL. (**B**) Working principles for the tricolour test strip platform for simultaneous qualitative and quantitative detection of TMS and MG.

TMS is a common fishery anaesthetic, but there are few rapid detection methods. In this work, we designed a tricolour immunochromatographic test strip with double T lines that allowed for the sensitive simultaneous detection of TMS and MG. As illustrated in Scheme 1, this system involved two-colour AuNP probes, a test strip with double test lines (TS-DTL) and a qualitative and quantitative detection system. First, two-colour AuNPs (red and blue) were synthesised and coated with mAbs to prepare probes specific for TMS and MG. Double test (T) lines were set up by spraying TMS-BSA on T_1_ and MG-BSA on the T_2_ line. The two-colour probes that were not bound with TMS or MG in the sample could be captured by TMS-BSA at the T_1_ line or MG-BSA at the T_2_ line, resulting in red or blue T lines. When the two-colour probes were captured by the IgG coated on the control (C) line, the C line appeared purple to the naked eye. According to the competitive principle, negative results appeared in tricolour, namely, the red T_1_, blue T_2_ and purple C lines. The higher the concentration of TMS and MG in the sample, the lighter the colour of the T_1_ and T_2_ lines of the test strip, while purple was always present as a quality control. Thus, a more accurate naked-eye qualitative detection was achieved by a distinct tricolour nanosensor. In addition, a sensitive simultaneous quantitative detection for TMS and MG was realised by using a mobile phone and ImageJ software to collect and analyse optical signals.

## 2. Materials and Methods

### 2.1. Reagents and Materials

Tricaine mesylate and malachite green standards were purchased from Dr. Ehrenstorfer GmbH (Dassel, Germany), and tetrachloroaurate (III) hydrate (HAuCl_4_·3H_2_O), trisodium citrate (Na_3_C_6_H_5_0_7_·12H_2_O), *N*, *N*-dimethylformamide (DMF), 3-aminobenzoic acid and hydroquinone (C_6_H_6_O_2_) were purchased from China National Medicines Co., Ltd. (Beijing, China). Bovine serum albumin (BSA) was purchased from Sigma–Aldrich (St. Louis, MO, USA), mouse antitricaine mAbs and mouse antimalachite green mAbs were purchased from Determine Biotech Co., Ltd. (Wuxi, China). The EDC (1-ethyl-(3-dimethylaminopropyl) carbonyl diamide hydrochloride), *N*-hydroxy succinimide (NHS), Tween-20 and BCA protein quantification kits were purchased from Sangon Biotech Shanghai Co., Ltd. (Shanghai, China). IgG secondary antibody, the test strip materials including the glass fibre membrane acting as the sample pad, the polyvinylchloride plate serving as backing support material, and filter paper used for the absorbent pad were purchased from Jin Biotech Co., Ltd. (Shanghai, China). Nitrocellulose (NC) membranes used to hold the antigen and anti-IgG antibody were provided by Sartorius Ltd. (Gottingen, Germany). The fish (*Ophiocephalus argus*) and shrimp (*Litopenaeus Vannamei*) were purchased from a local aquaculture market (Shanghai, China). Aquaculture water was collected from our campus (Shanghai, China).

### 2.2. Synthesis and Characterisation of Two-Colour AuNPs

The synthesis of red AuNPs was performed as described by Chen et al. [22]. Briefly, a clean flask containing 50 mL of 0.01% (*w*/*v*) HAuCl_4_ solution was heated to boiling under stirring. Subsequently, 1.35 mL of 1% (*w*/*v*) trisodium citrate solution was added and boiled until a wine-red colour appeared. Next, the heating was stopped, and the solution was cooled to room temperature. Finally, the obtained red AuNPs were stored at 4 °C before use.

Blue AuNPs were synthesised through seeding growth according to a method described by Peng et al. [21] with slight modifications. First, 160 μL of 1.2% (*w*/*v*) HAuCl_4_, 110 μL of 1% (*w*/*v*) trisodium citrate, and 1.2 mL of red-wine AuNP solutions were mixed in 50 mL of pure water under stirring. Next, the solution was heated to 50 °C. Subsequently, 0.84 mL of 30 mM hydroquinone solution was quickly added and mixed at room temperature for 30 min with constant stirring until the solution appeared a deep blue colour.

The size and morphology of the two-colour AuNPs were measured by a high-resolution transmission electron microscope (TEM; H-7800, Tokyo, Japan). The hydrodynamic size distribution of AuNPs was analysed by a scientific dynamic light scattering (DLS) nanoparticle analyser (Malvern Nano ZSE, London, UK). The UV–vis absorption spectra peaks of AuNPs were characterised on a microplate reader full wavelength scan (Bio-Tek ELISA, Winooski, VT, USA).

### 2.3. Preparation of AuNP Probes Specific for TMS and MG

Anti-TMS and anti-MG mAbs were modified on the surface of AuNPs according to the method of Wang et al. [23]. First, red AuNPs were adjusted to pH 7.5, and blue AuNPs were adjusted to pH 8.0 with 0.2 M K_2_CO_3_. Next, 2.5 µg of anti-TMS mAb and anti-MG mAb in 10 mM PBS buffer (pH 7.4) were separately added to the AuNP solution and mixed for 1 h at room temperature. Subsequently, 50 μL of 10% (m/v) BSA was added and kept for reaction for 1 h. The obtained precipitate was washed twice with 20 mM PBS containing 1% BSA and 0.5% Tween-20 (PBST) following centrifugation at 8000 rpm for 30 min. Finally, the precipitate was dissolved in 1 mL PBST containing 5% sucrose and 0.05% NaN_3_ and stored at 4 °C before use. The probe bound with TMS mAbs was red, while the probe coated with MG mAbs was blue.

### 2.4. Preparation of TMS-BSA and MG-BSA Antigens

The synthesis of TMS-BSA was performed using the mixed-anhydride method as described by Lei et al. [24]. Briefly, 12.5 μL of tributylamine and 6.8 μL of isobutyl chloroformate were added to 12 mg/mL 3-aminobenzoic acid in a DMF solution and mixed for 1 h. Subsequently, the mixture was added dropwise to 10 mg/mL BSA in 20 mM PBS and allowed to react for 12 h. Next, the solution was dialyzed with 20 mM PBS buffer for 3 days, during which the buffer was changed every 8 h. The above operation was performed at 4 °C. The concentration of TMS-BSA was quantified by a BCA kit.

The NHS/EDC chemical coupling method as described by Du et al. [25] was used for MG-BSA preparation. First, 100 μL of 31.0 mg/mL NHS and 28.8 mg/mL EDC were mixed with 50 μL of 25.0 mg/mL MG in DMF for 6 h in a microtube. Subsequently, 10 mg/mL BSA in 20 mM PBS was slowly added to the mixture and incubated for 4 h. The procedure of dialysis and concentration quantification of the MG-BSA solution was the same as that of the TMS-BSA preparation.

### 2.5. Construction of the Test Strip with Double T Lines (TS-DTL)

TS-DTL was prepared according to the method derived from Nardo et al. [26]. It comprised four parts, namely, a support plate, absorbent pad, NC membrane and sample pad, which were assembled layer by layer. A certain concentration of the TMS-BSA antigen was sprayed on the T_1_ line, while the MG-BSA antigen was sprayed on the T_2_ line, and 1.0 mg/mL goat antimouse IgG antibody was sprayed on the C line at 1 μg cm^−1^. After the NC membrane was dried for 3 h at 37 °C, a test strip with double T lines (TS-DTL) was assembled. Finally, each strip was cut into 5 mm diameter pieces, packaged and stored at room temperature.

### 2.6. Simultaneous Detection Procedure for TMS and MG

First, 90 μL of sample was mixed with 6 μL of red and 6 μL of blue AuNP probes in a microtube. Subsequently, 15 μL of PBS containing 0.2 g/mL BSA and 5% Tween-20 (*v*/*v*) was added and mixed for 30 s. The mixture was added to the sample pad of TS-DTL. After a reaction for 10~15 min, different colours at the T_1_, T_2_ and C lines were visible by the naked eye. Additionally, the optical grey signals in the T_1_, T_2_ and C lines could be scanned by a portable mobile phone and quantitatively analysed using ImageJ software. Additionally, the *B*/*B*_0_ ratio [27] between the sample (*B*) and negative control (*B*_0_) was calculated as an index for quantification analysis according to the following equation:

(1) $$B/B_{0}=\frac{GR_{T}/GR_{C}}{GR_{T_{0}}/GR_{C_{0}}}$$

where *B* is the ratio of the grey signals of the T and C lines of samples; *B*_0_ represents the ratio of the grey signals of the T and C lines of the negative control; $GR_{T}$ and $GR_{C}$ are the grey signals of the T and C lines of sample, respectively; and $GR_{T_{0}}$ and $GR_{C_{0}}$ are the grey signals of the T and C lines of the negative control, respectively. A low value for *B*/*B*_0_ (<0.2) indicated a strong positive result.

### 2.7. Detection Performance Evaluation of the TS-DTL Platform

#### 2.7.1. Specificity

Six common fishery anaesthetics, procaine hydrochloride, benzocaine, phenoxyethanol, propofol, eugenol and lidocaine, were used to evaluate the specificity of TS-DTL for TMS. The specificity of MG was evaluated by 6 dyes and structural analogues, including leuco-MG, patent blue, methyl blue, bright green, pararosaniline and rosolic acid. The concentrations of these chemical compounds were all 10 μg/mL in 20 mM Tris buffer (pH 6.8).

#### 2.7.2. Preparation of Real Samples Spiked with TMS and MG

A series of TMS and MG standards (0, 0.005, 0.025, 0.05, 0.1, 0.25, 0.5, 1, 2.5, 5, 10, and 25 μg) were individually spiked into 1 g of fish (*Ophiocephalus argus*) and shrimp *(Litopenaeus vannamei*) and 1 mL of aquaculture water. The sample preparation of fish and shrimp was prepared as follows. First, 2 g of muscle spiked with different TMS and MG (0.1, 1, 2 and 10 μg/g) was ultrasonically treated with 1 g of MgSO_4_, 0.4 g of primary secondary amine sorbent (Si (CH_2_)_3_NH(CH_2_)_2_NH_2_), 0.3 g of NaCl, 0.2 g of graphite carbon and 5 mL of acetonitrile for 10 min in a microtube. After centrifugation for 10 min at 8000 rpm, the collected supernatants were dried over flowing nitrogen at 60 °C. Next, the precipitates were dissolved in 1 mL Tris buffer containing 10% BSA (m/v) and 5% Tween-20 (*v*/*v*). The obtained resuspension solutions and the aquaculture water spiked with different concentrations of TMS and MG (0.1, 1, 2 and 10 μg/mL) were used as positive samples for TS-DTL and HPLC analysis. Fish, shrimp extraction and aquaculture water without TMS and MG standards were used as negative samples.

#### 2.7.3. Sensitivity and Accuracy Evaluation

Visual qualitative sensitivity was defined as the concentration when the T line was invisible. Quantitative LOD was calculated as the optical grey signals plus a threefold standard deviation of the negative samples [28]. Meanwhile, the spiked real samples described in Section 2.7.2 were used to evaluate the simultaneous detection sensitivity for TMS and MG. Additionally, the accuracy of the TS-DTL platform was evaluated by real samples and validated with the HPLC method. The recovery rate was calculated as the ratio between the measured concentrations of each sample and the spiked concentration.

### 2.8. HPLC Analysis of TMS and MG

The TMS and MG of spiked samples were quantified by an HPLC instrument (Agilent Infinity 1260, Santa Clara, CA, USA) including a DIKMA Diaminsil Plus C18-A column (250 × 4.6 mm^2^, 4.6 μm). The sample preparation was described in Section 2.7.2, and the resuspension solutions and the aquaculture water were filtered through a 0.22 μm polytetrafluoroethylene syringe filter before analysis. For the TMS detection, the mobile phase consisted of solvent A (acetonitrile) and solvent B (water) in a 7:3 ratio as isocratic elution. The HPLC conditions were as follows: flow rate 0.6 mL/min, column temperature 40 °C, injection volume 5 μL and wavelength 220 nm. For the MG detection, the mobile phase consisted of solvent A (5 mM ammonium acetate, pH = 4.5) and solvent B (methanol) in 4:6 as isocratic elution. The HPLC conditions were as follows: flow rate 0.6 mL/min, column temperature 60 °C, injection volume 5 μL and wavelength 618 nm. 

## 3. Results

### 3.1. Synthesis and Characterisation of Two-Colour AuNPs

**Figure 1:**
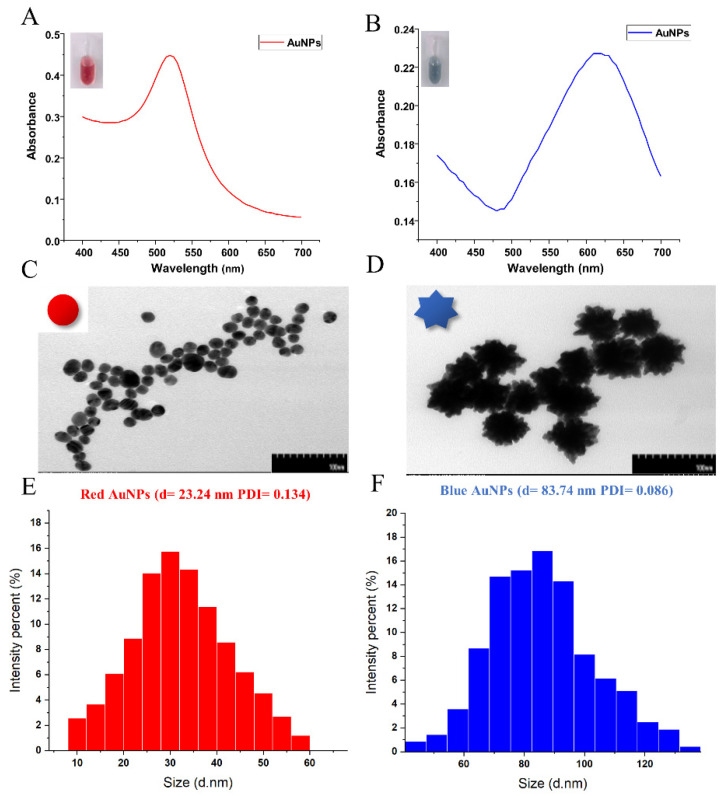
Characterisation of the optical properties, shapes and sizes of red and blue AuNPs. (**A**,**B**) UV–vis absorption spectra of two-colour AuNPs. Inset: the relevant photographic images of the synthesised AuNP solution. (**C**,**D**) TEM images. Inset: the structural diagrammatic presentation of synthesised AuNPs. (**E**,**F**) Hydrodynamic diameter. PDI indicates the polydispersity index.

AuNPs have morphology-/size-tunable localised surface plasmon resonance absorption (LSPR), which enables the synthesis of different sizes of AuNPs with unique colours. The red AuNPs, which were synthesised by the traditional citrate reduction method, had a maximal LSPR absorption at 520 nm (Figure 1A), whereas blue AuNPs had a maximal LSPR absorption at 620 nm (Figure 1B). According to the TEM images, the morphology of red AuNPs was spherical and smooth (Figure 1C), while blue AuNPs with larger sizes showed irregular shapes with multiple branches on the surface (Figure 1D). The DLS results showed that the average hydrodynamic diameter of the two AuNPs differed greatly. Blue AuNPs were 83.74 nm (Figure 1F), while red AuNPs were 23.24 nm (Figure 1E). The red AuNPs were similar to a previous report in morphology and size [24], while our blue AuNPs had a unique size compared with the 44.3 nm blue AuNPs reported by Wu et al. [17] and the 36.0 nm blue AuNPs reported by Peng et al. [21], which might be caused by different addition amounts of Au seeds and hydroquinone solution. Our results indicated that the two-colour AuNPs had a uniform particle size distribution and a significant difference in optical absorption wavelength, making them suitable as coloured probes to develop a simultaneous detection method.

### 3.2. Construction of the Tricolour TS-DTL for TMS and MG

#### 3.2.1. Optimisation of the Detection System

For a competitive ICTS, the stronger the positive result is, the lighter the colour of the T line. Thus, the bright T line colour is easy to observe, but it might lead to low sensitivity. Therefore, the effect of different conditions on the colour intensity of T lines was investigated. Herein, the optimal condition of TS-DTL was determined based on the fastest positive result given at a low concentration standard sample, and its *B*/*B*_0_ value should be less than 0.2.

**Figure 2:**
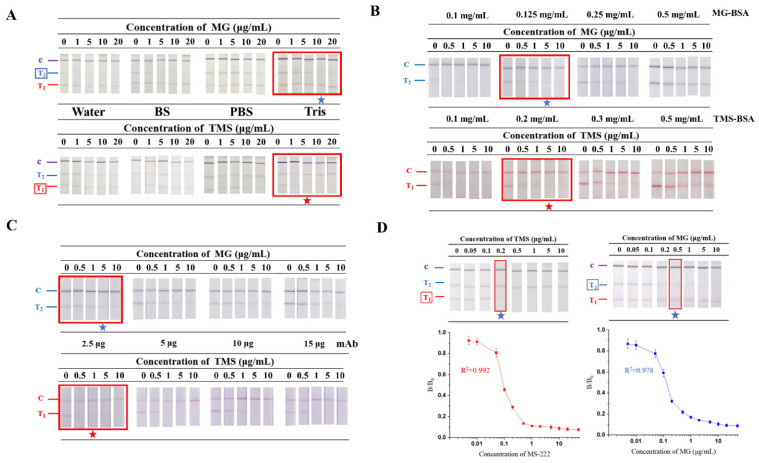
Optimisation results of different conditions. (**A**) Loading buffer solution. (**B**) Concentration of coating antigens of TMS-BSA and MG-BSA. (**C**) The amount of mAbs used to prepare AuNP probes for TMS and MG. (**D**) Single detection sensitivity of TS-DTL, (**left**) for TMS, (**right**) for MG. ★: Red and blue pentagrams indicate the qualitative limit of TMS and MG, respectively.

The principle of ICTS is based on the combination of mAbs and antigens, while different loading buffers affect capillarity action and their binding [24]. To reduce this interference, TS-DTL was optimised by three kinds of buffer (20 mM borate salt (BS), 20 mM PBS and 20 mM Tris), and water was used as a control group. TMS and MG standard dilutions (0, 1, 5, 10, 20 μg/mL) in different buffers were used as test samples. As shown in Figure 2A, when the antigen coating concentration of both the T_1_ and T_2_ lines was 0.5 mg/mL, clear tricolour (red T_1_, blue T_2_ and purple C) lines of TS-DTL were observed for the detection of TMS and MG standard samples (0~10 μg/mL) diluted in Tris and PBS buffer. Compared with PBS, the naked eye detection limit was 10 μg/mL, which was lower in the Tris buffer. Additionally, the *B*/*B*_0_ values of the corresponding TMS and MG concentrations were both less than 0.2, indicating that the Tris buffer was the optimal loading solution of TS-DTL for the detection of TMS and MG.

A fixed concentration of TMS-BSA (0.1, 0.2, 0.3, 0.5 mg/mL) on the T_1_ line and MG-BSA (0.1, 0.125, 0.25, 0.5 mg/mL) on the T_2_ line were employed to evaluate the optimal antigen coating concentrations. As shown in Figure 2B, with increasing antigen concentrations of TMS-BSA and MG-BSA, the signal intensities of the double T lines and C line were gradually enhanced. When the coating concentration of TMS-BSA at T_1_ was 0.2 mg/mL and the MG-BSA at T_2_ was 0.125 mg/mL, both the red T_1_ and blue T_2_ lines were invisible for 5 µg/mL TMS and MG. Additionally, the *B*/*B*_0_ values of TMS and MG were lower than 0.2, indicating that there were strong positive results at this concentration. Hence, 0.2 mg/mL TMS-BSA (0.2 μg cm^−1^ on T_1_ line) and 0.125 mg/mL MG-BSA (0.125 μg cm^−1^ on T_1_ line) were chosen as the optimum coating concentrations of the T lines.

Various amounts (2.5, 5, 10 and 15 μg) of anti-TMS and anti-MG mAbs were separately bound to the surface of red and blue AuNPs to prepare two-colour probes specific to TMS and MG. As represented in Figure 2C, the colour intensity of the test zones deepened gradually with increasing amounts of mAb coated on AuNPs. The colour intensity of the T_1_ line changed significantly with 2.5 μg and 5 μg mAb-coated probes when detecting the samples ranging from 0 μg/mL to 1 μg/mL TMS (Figure 2C). In addition, the *B*/*B*_0_ value at 1 μg/mL TMS was 0.183 for the 2.5 μg mAb-coated probe, indicating that it was a strong positive result. Meanwhile, the blue T line treated with 5 μg/mL MG using the 2.5 μg mAb-coated probe presented a lower *B*/*B*_0_ value (0.196). Thus, 2.5 μg mAb-coated probe was considered the optimal amount of mAbs labelled with AuNPs for both TMS and MG detection.

#### 3.2.2. Single Detection Sensitivity of TS-DTL for TMS or MG

To investigate the detection sensitivity under the optimised conditions, TMS and MG were separately detected, and their standard curves are shown in Figure 2D. It was obvious that the T_1_ line was invisible when the concentration of TMS was 0.2 μg/mL, while the T_2_ line entirely disappeared at 0.5 μg/mL MG. Therefore, the qualitative limit of TS-DTL was 0.2 μg/mL for TMS and 0.5 μg/mL for MG, which could meet the requirement of the TMS residue limit in the USA [3].

Competition inhibition curves for TMS and MG were established by plotting *B*/*B*_0_ values against analyte concentration. According to the linear regression equations from the TMS and MG standard curves, the LOD values of TMS and MG were calculated to be 8.9 ng/mL and 13.8 ng/mL, respectively. The quantitative sensitivity of our TS-DTL method for TMS was more than 10 times higher than the sensitivity (<0.1 μg/mL) reported by Lei et al. [24] based on colloidal gold-labelled ICTS. In addition, it was found that no cross-reaction between T_1_ and T_2_ was observed even at high concentrations (10 μg/mL) of TMS and MG, showing that the optimised tricolour TS-DTL was not only suitable to achieve accurate naked eye observation but also for the simultaneous detection of TMS and MG.

### 3.3. Performance Evaluation of TS-DTL

#### 3.3.1. Specificity

**Figure 3:**
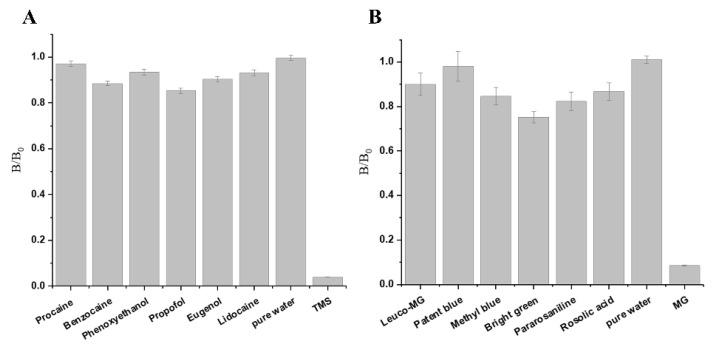
Specificity evaluation of TS-DTL for the detection of TMS and MG. (**A**) Specificity test of TMS and 6 other anaesthetics; (**B**) Specificity test of MG and 6 other dyes.

Six anaesthetics and six types of dyes were used to evaluate the specificity of TS-DTL for TMS and MG. As illustrated in Figure 3, both *B*/*B*_0_ ratios of TMS and MG were less than 0.1, indicating a strong positive result. However, the *B*/*B*_0_ ratios of the six anaesthetics and five dyes in addition to bright green were greater than 0.8, meaning they were negative results. The *B*/*B*_0_ of bright green was 0.752 (Figure 3B), indicating that the developed TS-DTL might have a slight cross-reaction with bright green. Overall, the developed TS-DTL was specific to TMS and MG.

#### 3.3.2. Simultaneous Detection Sensitivity of TMS and MG

**Figure 4:**
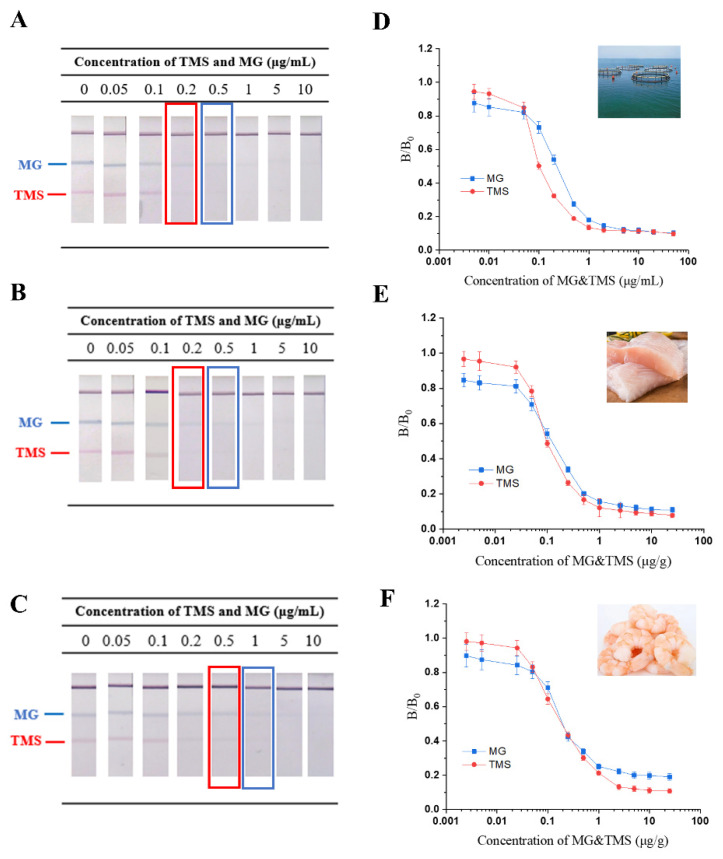
Simultaneous detection of TMS and MG in spiked samples with TS-DTL. Qualitative detection for aquaculture water (**A**), fish (**B**) and shrimp (**C**). Calibration curves of TMS and MG in aquaculture water (**D**), fish (**E**) and shrimp (**F**).

In general, TMS and MG are dissolved in water to keep aquatic products fresh and lively during aquaculture transportation. Therefore, the sensitivity of TS-DTL for the simultaneous detection of TMS and MG was investigated by three types of aquatic matrices (aquaculture water, fish and shrimp), which were spiked with TMS and MG at various concentrations (0~10 μg/g spiked in fish and shrimp, 0~10 μg/mL spiked in aquaculture water). As shown in Figure 4A–C, the colour of the T lines gradually faded with increasing concentrations of TMS and MG, while the red T_1_ line (TMS) was almost invisible at 0.2 μg/mL for aquaculture water and 0.5 μg/mL for fish and shrimp, which could be determined to be a qualitative limit. The blue T_2_ line (MG) disappeared at 0.5 μg/mL for aquaculture water and fish and 1.0 μg/mL for shrimp, which was determined to be qualitative sensitivity. For the aquaculture water, the quantitative sensitivity of TMS and MG was calculated to be 11.0 ng/mL and 47.0 ng/mL, respectively (Figure 4D). For the quantitative LOD of aquatic product samples, the detection limits of TMS and MG in fish were 29.6 ng/mL f and 82.8 ng/mL, respectively (Figure 4E), while the LOD of shrimp was 61.4 ng/mL for TMS and 152.4 ng/mL for MG (Figure 4F).

As shown in Table 1, the detection method for TMS mostly depended on instrumental analysis methods, such as HPLC and HPLC–MS/MS, which have good sensitivity but require professional technicians and are time-consuming. To the best of our knowledge, the ICTS method based on AuNPs for TMS did not give a clear quantitative LOD [24]. Herein, a fast and sensitive detection of TMS in three types of matrices (aquaculture water, fish and shrimp) was realised by the developed TS-DTL system, the TMS LOD (11.0–61.4 ng/mL) was over 16 times lower than the TMS residue limit of the USA (1 μg/mL). There have been various detection methods for MG, as shown in Table 1, and the LODs of MG have varied from 0.31 to 339.37 ng/mL based on different determination strategies [16,29,30,31,32,33]. The quantitative sensitivity of MG by the TS-DTL method was better than that of the ICTS based on orange carbon dots [16] and fluorescence method [30], but inferior to the ELISA [31] and colorimetry methods [32]. Overall, the TS-DTL system developed in this study could not only realise the simultaneous detection of TMS and MG but also be applicable to fish, shrimp matrices and related aquaculture water. The distinct tricolour system ensured the accuracy of visual observation, and the quantitative detection of the two substances within 20 min improved the detection efficiency.

#### 3.3.3. Accuracy Validation

The accuracy of the developed TS-DTL was evaluated through recovery experiments. Three spiked samples (aquaculture water, fish, shrimp) with concentrations of 0.1, 1, 2 and 5 μg/g TMS and MG were detected individually by the TS-DTL and HPLC methods.

As shown in Table 2 and Table 3, the average recovery of TS-DTL ranged from 85.1% to 108.6% for TMS and from 82.2% to 94.4% for MG, indicating acceptable accuracy. Moreover, it was found that the aquaculture water sample had the lowest coefficient of variation (CV < 10%) among the three types of samples. A higher CV (>7%) was found in fish and shrimp samples, especially at low concentrations (0.1 μg/mL), implying that the food matrix could affect the detection accuracy. The obtained recoveries and CVs by the developed TS-DTL were comparable to the ICTS methods reported previously [13,29]. In addition, the measurement results of three types of real samples by TS-DTL were in agreement with the data obtained by the well-accepted HPLC method, showing that the developed tricolour TS-DTLS could accurately and quantitatively detect TMS and MG in aquatic products and related aquaculture water.

## 4. Conclusions

In summary, a rapid tricolour TS-DTL system for the simultaneous detection of TMS and MG was established. Our methods provided a rapid and accurate visual identification of TMS and MG on site, which could minimise the risk of false judgement for various targets in the simultaneous presence of same-coloured T lines. A sensitive detection of TMS in aquatic products and aquaculture water was realised, the visual detection (0.5 μg/mL) of which could meet the requirements of the residue limit (1 μg/mL) of TMS in the USA. Meanwhile, the limit of quantitative detection of TMS (11.0~61.4 ng/mL ng/mL) was over 16 times lower than the residue limit of the USA. In addition, the tricolour test strip platform was rapid (~20 min, HPLC ~3 h, ELISA ~6 h) and stable, which can provide a multiple detection, on-site, rapid screening and identification tool for TMS and MG residues in aquatic products and aquaculture water.

## Figures and Tables

**Table 1:** Comparison of various methods for the quantitative detection of TMS and MG.

Analyte	Method	Label Material	LOD	Cost-Time	Sample	Reference
TMS	HPLC	/	25 ng/mL	6 h	fish	[7]
	HPLC–MS/MS	/	2.5 ng/mL	3.0 h	fish	[8]
	HPLC–MS/MS	/	0.01 ng/mL	1.0 h	water	[9]
	ICTS	AuNPs	<0.1 μg/mL	15 min	fish	[24]
	TS-DTL	Two-colour AuNPs	11.0 ng/mL	15~20 min	aquaculture water	This work
			29.6 ng/g		fish	
			61.4 ng/g		shrimp	
MG	ICTS	Orange carbon dots	58.4 ng/g	10 min	fish	[16]
	ICTS	AuNPs	1 ng/mL	8 min	fish	[29]
	Fluorescence	Se,N,Cl-doped carbon dots	339.37 ng/mL	1.5 h	fish	[30]
	assay					
	ELISA	/	0.31 ng/mL	6 h	fish	[31]
	Colorimetry	AuNPs	5 ng/mL	25 min	fish	[32]
	L-Aptamer	/	17.4 ng/mL	2 h	fish	[33]
	TS-DTL	Two-colour AuNPs	47.0 ng/mL	15~20 min	aquaculture water	This work
			82.8 ng/mL		fish	
			152.4 ng/mL		shrimp	

**Table 2:** Recovery evaluation of aquaculture water by the developed TS-DTL and HPLC methods.

Sample	Target	Spiked (μg/mL)		TS-DTL			HPLC	
			Found (μg/mL)	Recovery (%)	CV (%, *n* = 3)	Found (μg/mL)	Recovery (%)	CV (%, *n* = 3)
aquaculture water	TMS	0.1	0.094	94.2	7.51	0.11	110.0	3.56
		1	1.07	107.3	8.43	1.05	105.3	4.23
		2	1.91	95.5	6.44	2.06	103.0	3.51
		5	4.62	92.4	9.68	5.11	102.2	4.22
	MG	0.1	0.082	82.3	7.02	0.11	110.1	5.16
		1	0.92	92.4	7.93	1.01	101.4	3.92
		2	1.83	91.5	5.48	1.96	98.0	4.10
		5	4.67	93.4	8.70	4.79	95.8	3.95

**Table 3:** Recovery evaluation in aquatic products by the developed TS-DTL and HPLC methods.

Sample	Target	Spiked (μg/g)	TS-DTL				HPLC	
			Found (μg/mL)	Recovery (%)	CV (%, *n* = 3)	Found (μg/mL)	Recovery (%)	CV (%, *n* = 3)
fish	TMS	0.1	0.094	94.2	7.51	0.11	110.0	3.56
		1	1.07	107.3	8.43	1.05	105.3	4.23
		2	1.91	95.5	6.44	2.06	103.0	3.51
		5	4.62	92.4	9.68	5.11	102.2	4.22
	MG	0.1	0.082	82.3	7.02	0.11	110.1	5.16
		1	0.92	92.4	7.93	1.01	101.4	3.92
		2	1.83	91.5	5.48	1.96	98.0	4.10
		5	4.67	93.4	8.70	4.79	95.8	3.95
shrimp	TMS	0.1	0.085	85.4	13.27	0.102	102.0	4.76
		1	0.94	94.0	9.23	0.936	93.6	5.37
		2	2.07	103.5	8.63	1.89	94.5	4.87
		5	5.43	108.6	8.37	4.77	95.4	5.21
	MG	0.1	0.082	82.3	11.41	0.88	88.8	5.67
		1	0.88	87.6	12.56	0.92	92.0	4.88
		2	1.85	92.5	9.24	1.96	97.9	3.96
		5	4.24	84.8	7.31	4.22	84.3	4.63
